# Risk factors analysis and nomogram construction for postoperative pulmonary infection in elderly patients with hip fractures

**DOI:** 10.1007/s40520-023-02480-1

**Published:** 2023-06-26

**Authors:** Jingbiao Huang, Heng’an Ge, Xiaoping Zhu, Chao Xue, Qihang Su, Xujuan Chen, Biao Cheng

**Affiliations:** 1 Department of Sports Medicine, School of Medicine, Tongji Hospital, Tongji University, Shanghai, 200092 China; 2 Department of Nursing, School of Medicine, Shanghai Tenth People’s Hospital, Tongji University, Shanghai, 200092 China; 3 Department of Orthopaedic Surgery, School of Medicine, Shanghai Tenth People’s Hospital, Tongji University, Shanghai, 200092 China

**Keywords:** Hip fractures, Elderly patients, Pulmonary infection, Prediction model, Nomogram

## Abstract

**Purpose:**

The purpose of this study was to predict the probability of postoperative pulmonary infection in elderly patients with hip fractures by developing and validating a precise model.

**Methods:**

The clinical data of 1008 elderly hip fracture patients undergoing surgical treatment in Shanghai Tenth Peoples’ Hospital were retrospectively selected. A univariate analysis and multivariate regression were used to analyze the independent risk factors for postoperative pulmonary infection in elderly patients with hip fractures. A risk prediction model was established, and a nomogram was drawn. The area under the ROC curve and Hosmer‒Lemeshow test were used to evaluate the predictive effect of the model.

**Results:**

The multivariate regression analysis indicated that age > 73, time from fracture to surgery (d) > 4 days, smoking, ASA ≥ III level, COPD, hypoproteinemia, red cell distribution width > 14.8%, mechanical ventilation time > 180 min, and stay in the ICU were independent risk factors for postoperative pulmonary infection in elderly patients. The AUCs of the model were 0.891 and 0.881, 0.843, respectively, in the two verification groups. For the Hosmer‒Lemeshow test, the *P* values were 0.726 in the modeling group and 0.497 and 0.231 in the verification group (*P* > 0.05).

**Conclusion:**

Overall, this study uncovered different independent risk factors for postoperative pulmonary infection in patients with hip fractures. The nomogram can effectively predict the occurrence of postoperative pulmonary infection.

## Introduction

It was reported previously that the prevalence of hip fractures is increasing with the aging of the population. The literature [1] reported extremely high rates of mortality and disability, which are mainly due to postoperative complications, in elderly patients with hip fractures. Among these complications, lung infection is one of the most common complications, with an incidence ranging from 4.1 to 15.0% [2–4]. Chang [5] reported that a postoperative pulmonary infection can seriously affect the patient’s recovery, prolong hospital stays and even cause deaths in elderly patients with hip fractures. Odor [6] revealed that perioperative precise care markedly reduced the incidence of postoperative pulmonary infection. Thus, it is generally believed that early identification and intervention are of vital importance to prevent postoperative pulmonary infections.

In recent years, few studies have concentrated on risk factors analysis and the construction of prediction models for these patients. Furthermore, bias may exist in these available prediction models owing to the small number of cases, fewer included risk factors and validation at a single center in these studies, and the models from these studies are not suitable for clinical practical application and dynamic risk monitoring in elderly patients with hip fractures.

Therefore, the objective of this study was to adequately explore the independent risk factors for postoperative lung infection in elderly patients with hip fractures and to establish a predictive model based on these factors. This study provides an effective predictive tool for the early identification of high-risk patients and scientifically guides subsequent treatment.

## Materials and methods

### Research objects

Elderly patients who underwent hip fracture surgery in Shanghai Tenth People’s Hospital of Tongji University from May 2017 to May 2019 were selected as the research subjects. A total of 1008 patients met the inclusion criteria and were divided into a pulmonary infection group (87 patients) and a non-pulmonary infection group (921 patients) according to their postoperative condition.

The inclusion criteria were as follows: (1) based on the clinical symptoms, clinical signs, and imaging findings, a unilateral femoral neck fracture or a fracture of the intertrochanteric femur was diagnosed; (2) age ≥ 65 years; and (3) primary surgical treatment of hip fractures in the ipsilateral hip or lower extremities. The exclusion criteria were as follows: (1) a lung infection that was diagnosed before surgery, (2) combined with multiple fractures, and (3) death from a non-pulmonary infection postoperatively. The diagnostic criteria for pulmonary infection referred to the “Guidelines for the Diagnosis and Treatment of Hospital-Acquired Pneumonia and Ventilator-Associated Pneumonia in China” [7]. The diagnosis of lung infections in this study was given in consultation with qualified respiratory physicians. A total of 305 elderly patients with hip fractures who underwent surgery in Shanghai Tenth People's Hospital of Tongji University and 195 patients from Tongji Hospital of Tongji University were selected as the model validation groups with the same inclusion criteria as above.

This study was retrospective. Through the computer terminal, the hospital's digital medical record system was utilized to collect clinical data according to a questionnaire that was designed in advance. The data were collected by two researchers with more than 5 years of orthopedic work experience. For the individual missing values for the variables in the clinical data, the missing value was replaced by the mean of the column.

### Risk factors

A questionnaire for obtaining the clinical data of postoperative lung infections in elderly patients with hip fractures was compiled through a literature research and a panel meeting. In this questionnaire, we collected and analyzed the following risk factors for the research subjects: (1) basic information: age, sex, body mass index, smoking history, fracture type, time from fracture to surgery, number of preoperative comorbidities, antipsychotic drugs, and American Society of Anaesthesiologists (ASA) classification; (2) Whether there were underlying diseases before surgery: COPD, asthma, congestive heart failure, obstructive apnea syndrome, recent respiratory infections, history of other respiratory diseases, diabetes, cognitive impairment, history of stroke, malignant tumor history, ascites, and damaged sensory system; (3) Preoperative laboratory indicators (result of the first examination of admission): white blood cell count, hemoglobin, platelets, serum albumin, serum creatinine, blood urea nitrogen, and red cell distribution width (RDW); (4) Surgery-related factors: surgical method, anesthesia method, whether emergency surgery is available, and operative time; and (5) Perioperative treatment measures: Whether or not a gastric tube is indwelled, the duration of ventilatory support, whether to stay in the ICU, and whether to use long-acting neuromuscular blocking agents.

### Statistical analysis

The data were analyzed using SPSS 20.0 software. The counting data were statistically described using frequencies and percentages, and the two groups were compared using chi-square tests or Fisher’s exact probability method. The measurement data conforming to the normal distribution were expressed as the mean ± standard deviation and were compared using the *t* test. The skewed distribution measurement data were depicted using the median and interquartile range, and Wilcoxon rank-sum tests were applied for comparisons between the two groups. A risk factor analysis was performed using univariate and multivariate logistic regression analyses. A *P* value less than 0.05 was considered statistically significant. The variables of *P* minus 0.05 in the univariate analysis were included in the subsequent multivariate logistic regression analysis, and the risk variables eventually included in the predictive model were clarified by forwards stepwise regression analysis.

We selected collinearity diagnostics to conduct a multicollinear analysis between the independent variables and calculated the variance inflation factor (VIF) by EmpowerStats 2.0 (X&Y Solutions company, American) software. Eventually, an equation was constructed according to the partial regression coefficient corresponding to each variable, and a predictive model of postoperative complications of pulmonary infection was established for elderly patients with hip fractures. The risk assessment of postoperative pulmonary infection was simplified and visualized by drawing the nomogram. At the same time, the area under the ROC curve and the Hoster–Lemeshow test were used to validate the effect of the prediction model.

## Results

### General information

There were 1121 elderly hip fracture patients who were initially enrolled, of which 113 patients did not meet the requirements for inclusion. Eventually, 1008 patients were enrolled in the modeling group, and 87 patients (8.63%) developed postoperative lung infections. Furthermore, 305 elderly patients in Shanghai Tenth People's Hospital of Tongji University and 195 patients from Tongji Hospital of Tongji University were chosen as the model validation groups, with 22 patients (7.21%) and 15 patients (7.69%) developing postoperative lung infection.

### Univariate analysis

The results of the univariate analysis are shown in Table 1, and 18 statistically significant factors (*P* < 0.05) were filtered out, including age, history of smoking, time from fracture to surgery, number of preoperative comorbidities, being treated with antipsychotic drugs, ASA classification, COPD, recent respiratory infections, congestive heart failure, cognitive impairment, history of stroke, anemia, hypoproteinemia, high serum creatinine, high urea nitrogen, high RDW, check-in ICU and mechanical ventilation times.

**Table 1:** Univariate analysis of the risk factors of postoperative lung infection in elderly patients with hip fracture [*n* = 1008, cases (Percentage %)]

	Pulmonary infection group (*n* = 87)	Non-pulmonary infection (*n* = 921)	*Z*/*χ*^2^	*P* value
Basic information				
Age			23.429	< 0.001
≤ 73	5 (5.7)	242 (26.3)		
> 73	82 (94.3)	679 (73.7)		
Gender			1.632	0.196
Male	32 (36.8)	277 (30.1)		
Female	55 (63.2)	644 (69.9)		
BMI			3.614	0.060
< 18.5	20 (23.0)	127 (13.8)		
18.5–25	48 (55.2)	560 (60.8)		
25–30	18 (20.7)	210 (22.8)		
≥ 30	1 (1.1)	24 (2.6)		
Fracture type			0.020	0.886
Femoral neck fracture	37 (42.5)	399 (43.3)		
Intertrochanteric fracture	50 (57.5)	522 (56.7)		
Time from fracture to surgery (days)			28.836	< 0.001
≤ 4	38 (43.6)	656 (71.3)		
5 ~	31 (35.6)	202 (21.9)		
≥ 11	18 (1.8)	63 (6.8)		
Number of preoperative comorbidities			10.165	0.001
≤ 2	45 (51.7)	635 (68.9)		
> 2	42 (48.3)	286 (31.1)		
ASA classification			65.122	< 0.001
I	20 (23.0)	475 (51.6)		
II	27 (31.0)	360 (39.1)		
≥ III	40 (46.0)	86 (9.3)		
Being treated with antipsychotic drugs	8 (9.2)	13 (1.4)	14.137	< 0.001
History of smoking	42 (48.3)	222 (24.1)	21.479	< 0.001
Comorbidities				
COPD	23 (26.4)	4 (0.4)	96.750	< 0.001
Asthma	2 (2.3)	27 (2.9)	0.121	0.736
Recent respiratory infections	5 (5.7)	15 (1.6)	4.852	0.014
Congestive heart failure	14 (16.1)	24 (2.6)	24.476	< 0.001
Obstructive apnea syndrome	3 (3.4)	47 (5.1)	0.510	0.500
Diabetes	22 (25.3)	220 (23.9)	0.085	0.770
Cognitive impairment	13 (14.9)	50 (5.4)	9.368	0.001
History of stroke	45 (51.7)	276 (30.0)	16.199	< 0.001
Malignant tumor history	11 (12.6)	66 (7.2)	2.915	0.070
Ascites	0 (0)	1 (0.1)	0.181	1.000
Damaged sensory system	8 (9.2)	54 (5.9)	1.357	0.220
Preoperative laboratory indicators				
Anemia			39.302	< 0.001
Mild	43 (49.4)	352 (38.2)		
≥ Moderate	14 (16.1)	14 (1.5)		
RDW			6.179	0.008
> 14.8%	14 (16.1)	70 (7.6)		
≤ 14.8%	73 (83.9)	851 (92.4)		
Thrombocytopenia	7 (8.0)	41 (4.5)	1.928	0.138
Increased leucocyte count	24 (27.6)	181 (19.7)	2.881	0.081
Hypoproteinemia	14 (16.1)	5 (0.5)	49.648	< 0.001
High serum creatinine	24 (27.6)	100 (10.9)	16.478	< 0.001
High urea nitrogen	53 (60.9)	249 (27.0)	39.381	< 0.001
Surgery-related factors				
Surgical method			0.742	0.391
Proximal femoral nail anti-rotation	50 (57.5)	485 (52.7)		
Prosthetic replacement	37 (42.5)	436 (47.3)		
Operative time(min)			1.475	0.222
≤ 82	49 (56.3)	580 (63.0)		
> 82	38 (43.7)	341 (37.0)		
General anesthesia	78 (89.7)	870 (94.5)	2.777	0.075
Perioperative treatment measures				
Mechanical ventilation times (min)			34.747	< 0.001
≤ 180	67 (77.0)	885 (96.1)		
> 180	20 (23.0)	36 (3.9)		
Indwelling gastric tube	5 (5.7)	3 (0.3)	14.694	< 0.001
Check-in ICU	5 (5.7)	3 (0.3)	14.694	< 0.001

*ASA* American society of anesthesiologists, *COPD* chronic obstructive pulmonary disease, *RDW* Red cell distribution width

### Multivariate analysis

The risk factors that were statistically significant in the univariate analysis were included in the dichotomous unconditional multivariate logistic regression analysis, and the forwards stepwise method and likelihood ratio test were used for further screening of these risk factors. The logistics regression screened out 9 independent risk factors (*P* < 0.05*)* for postoperative complications of pulmonary infection in elderly patients with hip fractures, as shown in Table 2.

**Table 2:** Multivariate analysis of the risk of postoperative pulmonary infection in elderly patients with hip fracture

Regressor variable	Partial regression coefficient	Wald *χ*^2^	OR	95% CI	*P* value
Age	1.198	5.363	3.312	1.202–9.127	0.021
Time from fracture to surgery	0.631	8.014	1.880	1.214–2.910	0.005
History of smoking	1.285	16.847	3.616	1.957–6.680	< 0.001
ASA classification	0.720	11.840	2.054	1.36–33.096	0.001
COPD	4.455	39.689	86.014	21.513–343.897	< 0.001
Hypoproteinemia	3.258	20.218	25.988	6.282–107.514	< 0.001
RDW	1.629	12.099	5.097	2.036–12.759	0.001
Mechanical ventilation times (min)	2.035	21.349	7.651	3.227–18.138	< 0.001
Check-in ICU	1.207	14.675	3.343	1.803–6.199	< 0.001
Constant	− 5.951	107.054	0.003		< 0.001

*OR* odds ratio, *CI* confidence interval

These independent risk factors were diagnosed collinearly, and the variance inflation factors (VIFs) were all less than 10: 1.1, 1.0, 1.0, 1.1, 1.0, 1.0, 1.0, 1.1, and 1.2, indicating that there was no multiple collinearity among the nine independent risk factors.

### Model and nomogram development

**Fig. 1:**
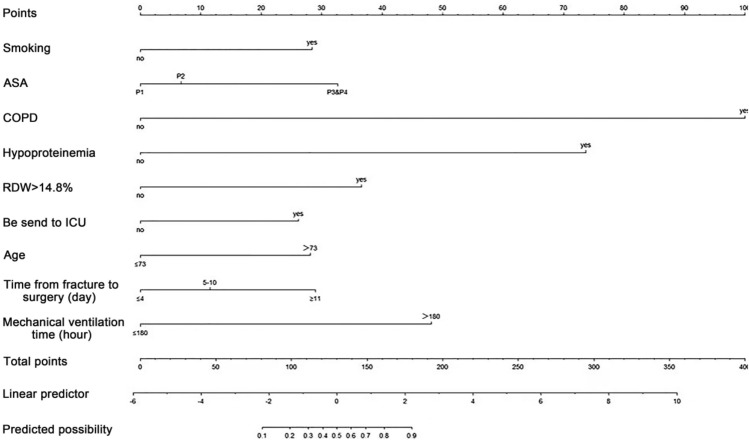
Nomogram for predicting the probability of postoperative pulmonary infection in the elderly patient with hip fractures

Based on the above independent risk factors and their corresponding regression coefficients, a predictive model of postoperative lung infection was constructed, and the formula is:

Z = − 5.951 + 1.198 × (age) + 0.631 × (time from fracture to surgery) + 1.285 × (history of smoking) + 0.720 × (ASA classification) + 4.455 × (COPD) + 3.258 × (hypoproteinemia) + 1.629 × (RDW) + 2.035 × (mechanical ventilation time) + 1.207 × (check-in ICU). A visual presentation of the simplified nomogram is depicted in Fig. 1.

### Model validation

**Fig. 2:**
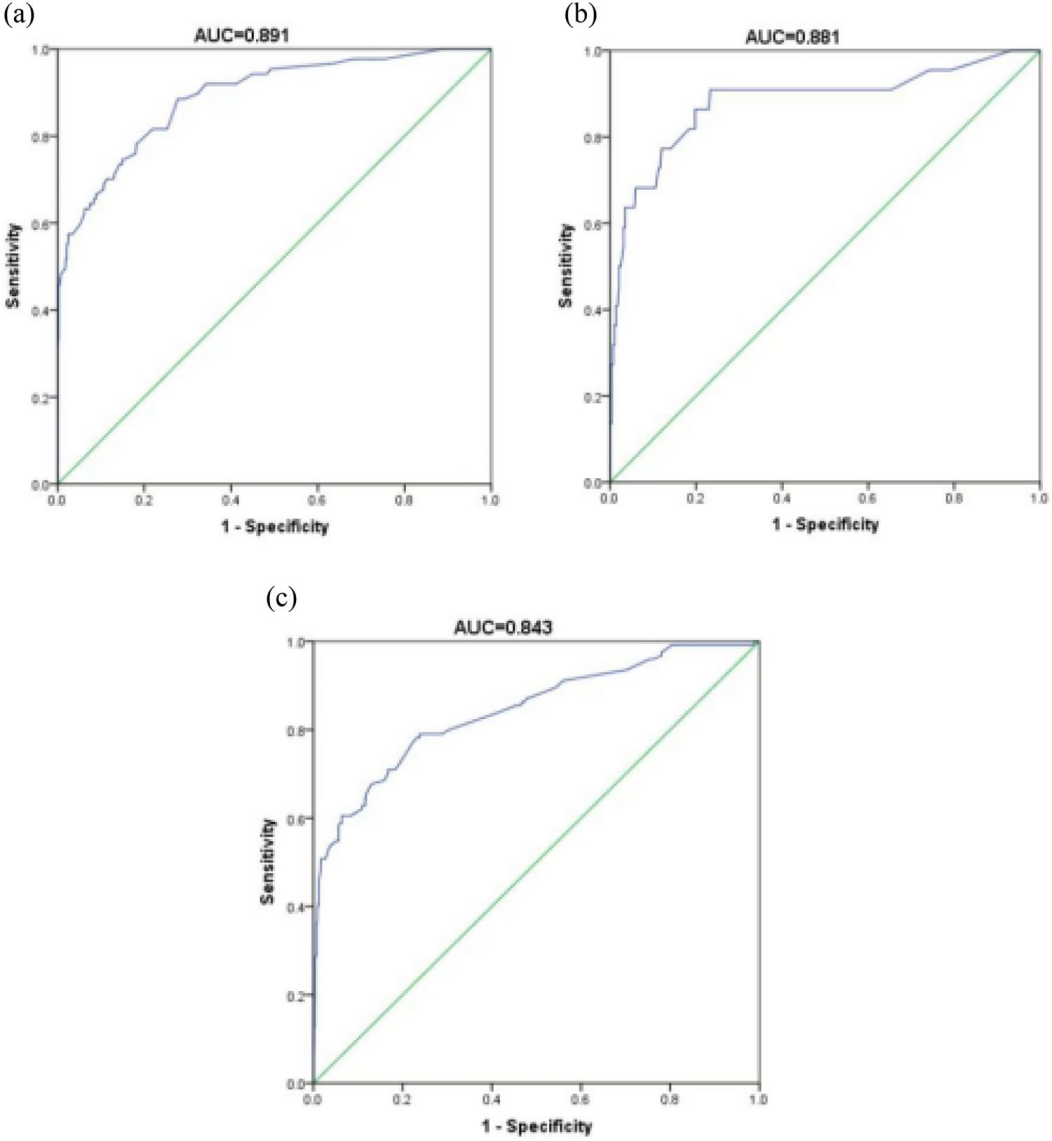
ROC curves for validating the discrimination power of the nomogram. **a** Modeling group **b** Validation group 1 **c** Validation group 2

The validation of the model needs to evaluate the discrimination and calibration in two dimensions. In this study, we evaluated the sensitivity and calibration of the predictive model by drawing ROC curves of the predicted probability and conducting the Hosmer‒Lemeshow test. The preliminary results showed that the area under the ROC curve for the risk of postoperative pulmonary infection in the modeling group was 0.891, and the 95% CI was 0.853–0.930 (*P* < 0.001). The sensitivity was 0.828, the specificity was 0.786, and the Youden index was 0.614. As shown in Fig. 2, the area under the ROC curve in validation Group 1 (the same center) was 0.881 and 0.843 in validation Group 2 (another center), which suggested high discrimination of this model.

Validation g It was also shown that the *P* values of the Hosmer‒Lemeshow test in the modeling group and the groups were 0.726, 0.497 and 0.231 (*P* > 0.05), respectively, suggesting that there are no significant differences between the actual and predicted values.

## Discussion

In this study, we retrospectively evaluated 1008 elderly patients with hip fracture postoperatively to screen for independent risk factors associated with postoperative pulmonary infections. The results of univariate and multivariate analyses suggested that age, time from fracture to surgery, history of smoking, ASA classification, COPD, hypoproteinemia, RDW, mechanical ventilation time, and check-in ICU were independent risk factors for postoperative complications of pulmonary infection in elderly patients with hip fractures. The risk prediction model for postoperative pulmonary infection in elderly hip fracture patients was constructed through an extensive data analysis. A subsequent validation of the results due to obtaining a similar area under the ROC curve indicated that the risk prediction model accurately identifies elderly hip fracture patients who have postoperative pulmonary infections. In addition, the *P* values in the test of calibration were 0.726, 0.497, and 0.231 and were significantly greater than 0.05, proving that the probability of the model predicting the occurrence of postoperative pulmonary infection was close to the actual observed probability. A visual presentation of the nomogram was also established to facilitate clinical application.

Previously, it was recognized that COPD is an independent risk factor for postoperative pulmonary infection in elderly patients with hip fractures. COPD is more commonly seen in elderly patients and is characterized by persistent or progressive restriction of respiratory airflow. Galizia G et al. [8] concluded that long-term mortality was higher in elderly patients with than in those without COPD. Besides, Ekstrom et al. [9] proved that the incidence of postoperative pulmonary infections was significantly higher in patients with preoperative comorbid COPD. The results of the present study also showed that having COPD as a preoperative comorbidity was highly positively associated with the risk of postoperative complications of pulmonary infection (OR = 86.014), and its OR was significantly higher than the remaining influencing factors. On the one hand, the structure and function of the COPD patient’s lungs and thorax are altered owing to the long-term effects of COPD, which results in reduced compliance, abnormal pulmonary blood flow, irreversible lung injury [10], and severe frailty [8]. On the other hand, patients with comorbid COPD mostly have a long history of smoking, which further increases the risk of postoperative lung infection. Therefore, to optimize lung function, targeted interventions should be provided to these high-risk patients preoperatively, such as training with balloon blowing, lip retraction breathing and education about smoking cessation [11]. Meanwhile, nebulized inhalation should be available to promote sputum dilution and expulsion [12].

Serum albumin is the most commonly used serum marker to evaluate patients’ nutritional status in clinical practice. A serum albumin level less than 30 g/L is defined as hypoproteinemia, suggesting that the patient is malnourished. Bohl et al. [13] verified that preoperative combined hypoproteinemia in elderly hip fracture patients was an independent risk factor for postoperative complications of pulmonary infection, which is consistent with the results of this study. A large amount of protein is required for fracture healing and muscle strength recovery, and protein deficiency may lead to reduced muscle strength and limb function, which ultimately affect fracture healing and prolong bed stay, causing an increased risk of crush pneumonia [14]. In addition, a low serum albumin level results in a decrease in plasma colloid osmotic pressure and an increase in the amount of interstitial fluid, which may further develop into pleural and pulmonary infections [15]. Weimann et al. [16] suggested that patients with preoperative malnutrition should be operated on after correcting their altered nutritional status, and early surgical treatment has been shown to have more advantages in elderly patients with hip fractures [17]. Consequently, several studies [16, 18, 19] have indicated that elderly hip fracture patients who are undernourished or at risk of malnutrition should be given high-protein nutritional supplements preoperatively. This intervention can significantly reduce the incidence of multiple postoperative complications, such as pulmonary infection.

It is well established that the preoperative physical status exerts an important impact on the prognosis of elderly patients with hip fractures. In this study, age was also shown to be an independent risk factor for postoperative pulmonary infection (*P* = 0.021). Yanagi S et al. [20] agreed that the respiratory system of elderly patients undergoes various changes accompanied by the progressive decline of organ functions with age, including reduced chest wall compliance, weakened respiratory muscle strength, decreased cilia clearance, and a weakened cough reflex. However, age is too homogeneous to act as a separate predictor of postoperative pulmonary infection because of its relevance to the number of concomitant diseases, and it cannot be easily distinguished from diseases of the lung. Previously, Khan MA et al. [21] and Holt G [22] believed that elderly hip fracture patients with poor underlying physical conditions should be appropriately treated preoperatively for a period of time until their physiological indicators maintained stability, which means a longer preoperative waiting time. Nevertheless, the above results from this study showed that the incidence of postoperative pulmonary infection was lowest in elderly hip fracture patients with a time from fracture to surgery ≤ 4 days and was highest in patients with a time from fracture to surgery ≥ 11 days. Other studies [23, 24] similarly demonstrated that shortening the preoperative waiting time can significantly reduce the incidence of postoperative complications (including pulmonary infections) and mortality rate. According to international guidelines, it is also recommended that patients undergo surgical treatment within 48 h after a fracture occurs [25, 26]. Hence, early adjustment of relevant physiological indicators and timely surgery (≤ 4 days) are required for this group of patients. In addition, the ASA classification is a vital tool to assess the intraoperative and postoperative risk of patients according to the severity of their preoperative comorbidities [27]. Folbert et al. [28] proposed that elderly hip fracture patients with ASA grade ≥ III had significantly more postoperative complications than those with ASA grades I–II. In this study, we also proved that postoperative complications of pulmonary infection in elderly hip fracture patients were positively correlated with ASA grade, and the risk of postoperative pulmonary infection was twice as high in patients with ASA grade ≥ III than in patients with ASA grade < III. In conclusion, for patients with unstable physical conditions, multidisciplinary teams should be established by medical staff to adjust and treat conditions such as heart failure and electrolyte disturbances as soon as possible. Surgical treatment should not be postponed for unrealistic goals.

The prediction model and nomogram constructed in this paper have wide applicability. First, doctors in the outpatient area can evaluate elderly patients with hip fractures and assess the risk of postoperative pulmonary infection to decide if surgery is needed. This model can be an important reference indicator for surgeons to screen high-risk populations and choose surgical plans, including surgical methods, operation time and the time from fracture to surgery. For example, hemiarthroplasty tends to be a better surgical method for high-risk elderly patients with femoral neck and intertrochanteric fractures. The advantages of hemiarthroplasty lie in that it requires less operation time and allows early ambulation of patients. Additionally, based on this prediction model, the inpatients’ risk of perioperative pulmonary infection can be dynamically assessed, and effective preventive nursing measures should be taken in advance to reduce the incidence of lung infection and improve patients' long-term quality of life.

Several shortcomings in this study should be mentioned apart from retrospective case‒control studies. First, the patients’ discharge date was taken as the observation endpoint in this study, and any lung infection developing after discharge postoperatively was not included. As a result, the obtained incidence of postoperative lung infection may be lower than the actual incidence. In addition, further research for clinical application and multicener promotion is necessary to further adjust and optimize the values of the model and improve the accuracy of the model for forecasting.

## Conclusion

In elderly hip fracture patients, age > 73 years, time from fracture to surgery > 4 days, history of smoking, ASA grade III and above, combined COPD, combined hypoproteinemia, RDW > 14.8%, admission to ICU, and long duration of mechanical ventilation were independent risk factors for postoperative pulmonary infection. The risk prediction model constructed in this study presented good discrimination and calibration and can be widely used in clinical prediction and therapeutic intervention.

## Data Availability

The datasets used or analyzed during the current study are available from the corresponding author on reasonable request.
